# Classification of Visually Induced Motion Sickness Based on Phase-Locked Value Functional Connectivity Matrix and CNN-LSTM

**DOI:** 10.3390/s24123936

**Published:** 2024-06-18

**Authors:** Zhenqian Shen, Xingru Liu, Wenqiang Li, Xueyan Li, Qiang Wang

**Affiliations:** 1 School of Electronic and Information Engineering, Tiangong University, Tianjin 300387, China; liuxingru1234@163.com (X.L.); liwenqiang4519@outlook.com (W.L.); 2230070949@tiangong.edu.cn (X.L.); 2 Faculty of Psychology, Tianjin Normal University, Tianjin 300387, China; wangqiang113@gmail.com

**Keywords:** visually induced motion sickness, phase-lock value, CNN-LSTM

## Abstract

To effectively detect motion sickness induced by virtual reality environments, we developed a classification model specifically designed for visually induced motion sickness, employing a phase-locked value (PLV) functional connectivity matrix and a CNN-LSTM architecture. This model addresses the shortcomings of traditional machine learning algorithms, particularly their limited capability in handling nonlinear data. We constructed PLV-based functional connectivity matrices and network topology maps across six different frequency bands using EEG data from 25 participants. Our analysis indicated that visually induced motion sickness significantly alters the synchronization patterns in the EEG, especially affecting the frontal and temporal lobes. The functional connectivity matrix served as the input for our CNN-LSTM model, which was used to classify states of visually induced motion sickness. The model demonstrated superior performance over other methods, achieving the highest classification accuracy in the gamma frequency band. Specifically, it reached a maximum average accuracy of 99.56% in binary classification and 86.94% in ternary classification. These results underscore the model’s enhanced classification effectiveness and stability, making it a valuable tool for aiding in the diagnosis of motion sickness.

## 1. Introduction

In recent years, virtual reality (VR) has seen widespread adoption across various domains. VR technology immerses users in computer-simulated environments, enhancing their sense of presence by presenting three-dimensional characters, scenes, or objects [1,2]. However, a common issue experienced by many users is the onset of discomfort, including symptoms such as disorientation, nausea, sweating, and dizziness, collectively known as visually induced motion sickness (VIMS) [3]. VIMS occurs due to a discrepancy between visual motion cues and vestibular motion signals perceived by the inner ear vestibular system [4,5]. For most VR users, these symptoms manifest within approximately 15 min of use and can persist for a significant duration, posing a substantial obstacle to the advancement of VR technology [6,7]. Consequently, there is a critical need to explore effective methods for detecting VIMS and preemptively identify its onset to mitigate or prevent severe occurrences.

Currently, researchers have focused on VIMS detection using physiological indicators such as EEG, eye movement, and heart rate [8,9,10,11,12,13]. Among these indicators, EEG signals have garnered significant attention due to their rich data and information content. Researchers utilize changes in subjects’ EEG characteristics to characterize motion sickness [14]. For instance, Krokos et al. [8] quantified VR motion sickness using EEG and identified significant correlations between δ-, θ-, and α-waves and motion sickness, though they did not analyze the correlation with γ-waves. Lin et al. [15] observed enhanced γ-wave energy in subjects experiencing motion sickness. Chen et al. [16] noted a decreasing trend in the power spectral density of α-waves in the parietal and motor regions of motion sickness subjects, while the θ- and δ-wave power spectral density increased in the occipital regions. However, these studies primarily focused on the power spectral density of EEG signals and did not consider other EEG parameters.

Current research on EEG primarily utilizes time domain analysis and frequency domain analysis for feature extraction. In time domain analysis, features such as mean and peak values are extracted as characteristic parameters from the EEG signals [17]. In frequency domain analysis, features such as the centroid frequency are extracted from EEG signals [18]. The brain is an extremely complex network system, and extracting only time or frequency domain features from EEG signals may not adequately capture the coupling relationships between different brain regions.

Brain functional connectivity is a metric used to measure the relationships between different regions of the brain, facilitating a better understanding of the coupling relationships among these areas [19]. Common indices of functional connectivity include the Pearson correlation coefficient (PCC) [20], the phase lag index (PLI) [21], and the phase-locking value (PLV) [22]. Among these, the phase-locking value (PLV) is used as an index to measure the phase synchrony of signals between different brain regions and is commonly utilized to analyze interactions and synchrony between various areas [23]. Di Biase et al. [24] found that, in both resting and active states, healthy controls (HC) exhibited a higher delta-band PLV connectivity during motion compared to patients with Parkinson’s disease (PD). However, it remains unclear whether the PLV functional connectivity matrix can capture the functional connections between different brain regions in subjects with visually induced motion sickness and accurately differentiate various states of this condition. Further research is needed to clarify these aspects.

In the field of visually induced motion sickness detection algorithms, researchers have conducted studies using machine learning algorithms. Zhu et al. [25] utilized wavelet transform to extract EEG features related to motion sickness. They combined these features with SVM, achieving a two-classification accuracy of 79.25%, and a three-classification accuracy of 70.37%. Hua et al. [26] investigated EEG signals during resting virtual reality motion sickness. By decomposing EEG signals from FP1, FP2, F8, T7, and T8 electrodes using variational mode decomposition, they extracted sample entropy, permutation entropy, and center frequency, achieving a classification accuracy of 98.3% with an SVM classifier. The aforementioned studies utilized various machine learning algorithms to classify virtual reality motion sickness. However, the former exhibited suboptimal accuracy in classification, while the latter was limited to binary classification problems and did not extend to three-class or multi-class tasks. Additionally, classification was only performed on resting state virtual reality motion sickness. Given the difficulties traditional machine learning algorithms face with nonlinear problems and their limited capacity to handle large datasets, the introduction of deep learning algorithms has proven to be an effective solution. Yildirim [27] reviewed research on detecting motion sickness from EEG signals using deep learning frameworks, noting that deep learning algorithms, such as DNNs, CNNs, and RNNs, could effectively classify motion sickness. However, the provided classification models are relatively simplistic. Similarly, Hua et al. [28] demonstrated that the best regression results prior to using LSTM/BiLSTM networks were obtained through CNNs, which integrated rhythm information from all electrodes. Yet, that paper only used EEG data as the model input, which is not interpretable, and there is no classification study.

Based on the above analysis, we aim to utilize the PLV functional connectivity matrix as a feature parameter to study the functional connectivity characteristics between different brain regions under VIMS conditions across different frequency bands. We plan to construct a CNN-LSTM hybrid model, leveraging the brain’s functional connectivity features and deep learning algorithms to enhance the detection effectiveness of visually induced motion sickness.

## 2. Materials and Methods

### 2.1. Participants

A total of 28 college students with normal vision or corrected vision were recruited as subjects for this study. They were carefully screened to exclude any vestibular disorders, gastrointestinal diseases or conditions that could potentially cause vertigo. Additionally, these subjects had no prior exposure to similar experiments or knowledge of the experimental procedures. To ensure data reliability, subjects were instructed to refrain from alcohol consumption for 24 h prior to the experiment and maintain a positive mental state. Due to factors such as excessive noise and missing crucial information, three participants were excluded from the final analysis. Thus, the data from 25 subjects were retained, comprising 20 males and 5 females, with an average age of 21.32 ± 2.27 years.

### 2.2. Experiment Platform Construction

**Figure 1:**
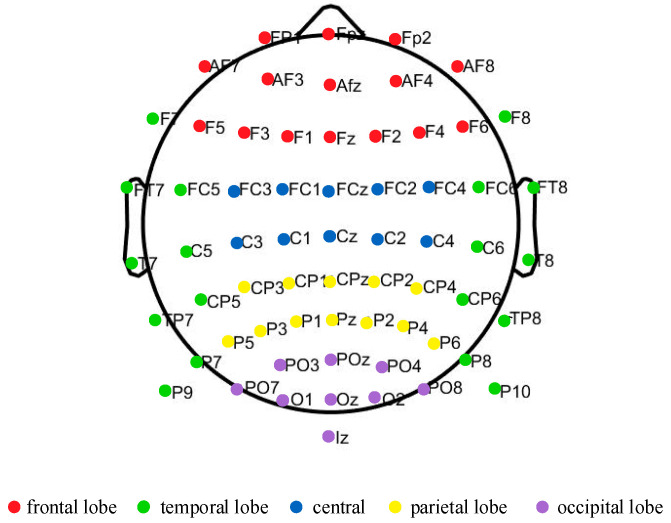
Electrode position diagram.

The experiments were conducted utilizing an EEG device (64 channels) from BioSemi, the Netherlands, for EEG data acquisition. The device had a sampling frequency of 2048 Hz and the electrodes were placed according to the internationally recognized 10–20 system. For the purpose of data analysis, the 64 channels were categorized into five regions: the frontal lobe, temporal lobe, central, parietal lobe, and occipital lobe, as shown in Figure 1. Additionally, the HTC VIVE pro 1.0 head-mounted display, along with its accompanying computer system, served as the platform for virtual reality. Steam VR software 2.5.5 facilitated the seamless interaction between the user and the virtual reality environment. In this study, the video titled “Desert Ride Coaster”, which features a 90 s automatic cycle playback, was chosen as the stimulus to induce visually induced motion sickness among users. Prior studies have confirmed that this video can rapidly trigger VIMS among users [29].

### 2.3. Experimental Procedure

**Figure 2:**
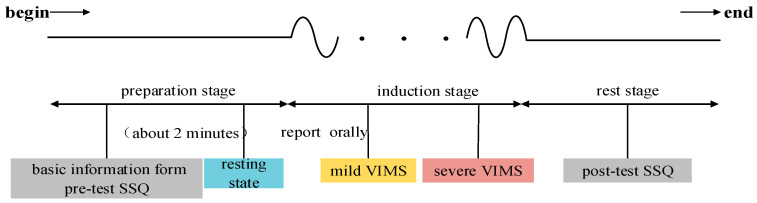
Flow chart of VIMS evoked experiment.

The whole VIMS induction experiment is divided into the preparation stage, induction stage, and the rest stage, and the experimental flow is illustrated in Figure 2. During the preparation stage, the participants were briefed on the whole experimental process and precautions, and signed the informed consent. Subsequently, the subjects filled out the basic information form and the pre-test SSQ (Simulator Sickness Questionnaire). The SSQ scale is used as a subjective measurement of virtual reality motion sickness. It is a motion sickness assessment scale [30] developed by researcher Kennedy and others based on a series of experiments. Its validity and reliability have been established and used in many related studies. The scale requires subjects to fill out the questionnaire based on their true feelings at the time of the experiment, and the results of the questionnaire are be used as a benchmark for visually induced motion sickness. The questionnaire consists of 16 different symptoms, each of which is categorized into 4 levels of symptom degree: none, mild, moderate, and severe; and 3 measured weight values for ocular discomfort: oculomotor (O), disorientation (D), and nausea (N). The weights of these symptoms can be used to calculate the final score of the SSQ.

After installing EEG electrodes and applying conductive paste, the subjects wore the VR head display. First, the subjects’ 2 min resting state EEG data were collected. Next, at the VIMS induction stage, the subjects were shown the video *Desert Ride Coaster*. With the increase in watching time, the degree of motion sickness of the subjects would gradually increase. During this process, if the subjects felt a change in the degree of motion sickness, they needed to immediately report the grade of motion sickness at that time orally, including whether it was mild and severe. The experimenters synchronously recorded the time nodes of the two grades of motion sickness of the subjects. When the subject could not tolerate it, the motion sickness level at that moment was recorded as severe, and the video playback was stopped immediately. During the resting phase, the VR headset and EEG acquisition equipment were removed for the subjects, and the subjects were asked to fill in the post-test SSQ in due time.

### 2.4. EEG Signal Preprocessing

The raw EEG data were first preprocessed using the EEGLAB toolkit, and for the convenience of subsequent analyses, the electrodes with poor signals (PO4, O2, PO8, P10, TP8, F6, FP2, FPz, POz, P2, P1, PO3) were excluded from the EEG signals of all subjects, so that 52 valid channels were retained. The signals were filtered with a 0.5–45 Hz band-pass filter and a 49–51 Hz notch filter to eliminate power frequency interference. Then, the sampling frequency was reduced to 1024 Hz, and the whole brain average was used for re-reference. Finally, artifacts such as EMG and EOG were eliminated through independent component analysis (ICA). Then, the EEG signals of 25 subjects after pretreatment were divided into frequency bands: delta band (0.5–4 Hz), theta band (4–8 Hz), alpha band (8–14 Hz), beta band (15–30 Hz), gamma band (30–45 Hz), and full band (0.5–45 Hz).

### 2.5. PLV Functional Connectivity Matrix Construction

The PLV serves as a metric to evaluate the strength of functional connectivity between two or more electrode signals by calculating their phase difference, thereby analyzing the synchronicity of brain functional connectivity.

Assuming that the EEG signals of any two electrodes are x(t) and y(t), the PLV can be calculated by Equations (1) and (2).

(1) $$\Delta \varphi_{n}(t)=\left|\varphi_{x}\left(t\right)-\varphi_{y}\left(t\right)\right|$$

(2) $$PLV\left(t\right)=\frac{1}{N}\left|\sum_{n=1}^{N}e^{j\left(\Delta \varphi_{n}\left(t\right)\right)}\right|$$

In Equation (1), $\Delta \varphi_{n}(t)$ represents the instantaneous difference between signals x and y at time t. In Equation (2), N denotes the total number of sampling points utilized in the computation. The PLV ranges from 0 to 1, providing a quantitative measure of phase synchronization between the signals. When PLV = 0, it indicates that there is no phase synchronization, and that the two signals are independent, with no obvious synchronization. On the contrary, if the PLV tends to 1, it indicates that the phase coupling of the two signals is stronger, and the degree of synchronization is higher.

**Figure 3:**
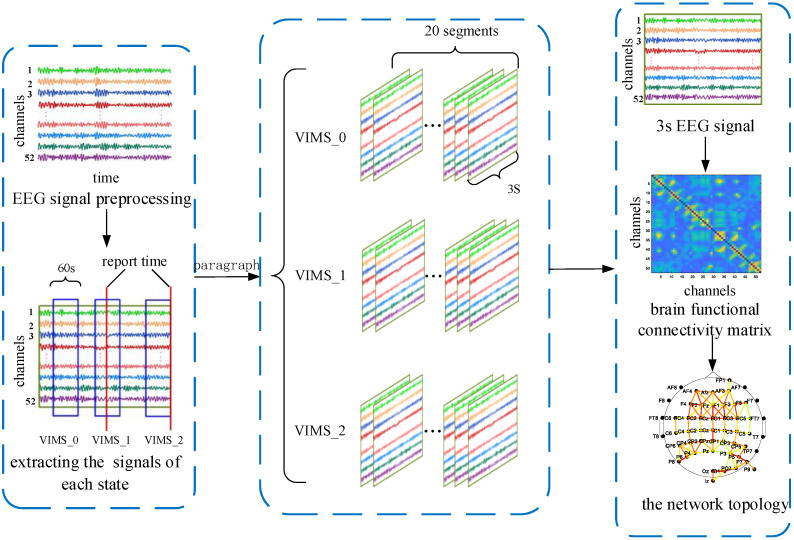
Flow chart of brain functional connectivity matrix feature extraction.

On the basis of the above EEG signal preprocessing, after extracting the EEG signals of each state, signal segmentation, calculating the PLV, and constructing the brain functional connectivity matrix, the network topology was finally mapped, as shown in Figure 3.

In Figure 3, we illustrated the process of analyzing the EEG signal within a certain frequency band as an example; the 60 s EEG signal in the resting state (VIMS_0) was first extracted. Subsequently, the 60 s signal (30 s before and after) was extracted at the moment of the light halo state (VIMS_1) report as the light halo signal; and since the experiment was stopped immediately after the second report, the 60 s signal before its verbal report was extracted as the heavy halo state (VIMS_2) signal.

Next, given that the lowest frequency of the EEG signal is 0.5 Hz, to ensure that each segment contains enough data to reflect the entire cycle of the lowest frequency component, the EEG signals of each frequency band were segmented with a 3 s non-overlapping time window, so that the EEG signals of each subject in each halo state were divided into 20 segments for each frequency band. Finally, the PLV functional connectivity matrix was calculated separately for each EEG segment, and the size of each PLV functional connectivity matrix was 52 × 52 (number of channels × number of channels), and the corresponding network structure topology was plotted.

### 2.6. Classification Modeling

**Figure 4:**
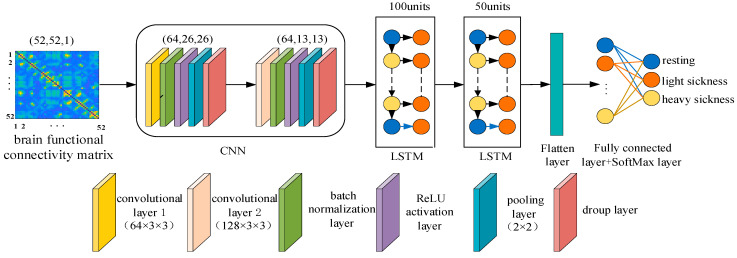
VIMS classification model based on PLV functional connectivity matrix and CNN-LSTM.

Considering the spatial feature extraction advantages of the convolutional layer in the CNN network model and the temporal modeling advantages of the LSTM network model, this paper proposes a VIMS classification model based on the PLV functional connectivity matrix and CNN-LSTM, which can capture the local features,› as well as retain the long-term dependencies when processing EEG signals. Its network model structure is shown in Figure 4.

The PLV functional connectivity matrix (52 × 52) served as the input to the CNN-LSTM model, which sequentially traverses through convolutional block 1 and convolutional block 2, resulting in output dimensions of (64, 26, 26) for convolutional block 1 and (128, 13, 13) for convolutional block 2. Each convolutional block contains a convolutional layer, a batch normalization layer, a ReLU activation layer, a pooling layer and a dropout layer. The convolutional layer is mainly used to extract spatial features, the size of the convolutional kernel is 3×3, the step size is 1, and the padding is 1. The batch normalization layer speeds up the training and improves the generalization ability of the model. The ReLU activation layer applies nonlinearities and sparsity to the network structure and prevents the gradient from vanishing. The expression for the ReLU function is

(3) $$f\left(x\right)=Max\left(0,x\right)=\left\{\begin{array}{c} 0\;x\leq 0\;\\ x\;x>0 \end{array}\right.$$

The pooling layer employed a maximum pooling layer with a size of 2 × 2, effectively reducing the model’s parameters and memory requirements; the dropout layer “drops” neurons in the network with a certain probability during the network training process, thereby enhancing the model’s generalization ability, and in this experiment, the first layer of the dropout was set to 0.5, and the second layer of the dropout was set to 0.3. Next, the relevant features extracted by the CNN are fed into the two-layer LSTM network to obtain all the temporal features of the motion sickness state in turn. The first layer LSTM had 100 hidden units, and the second layer LSTM had 50 hidden units. There were three gates in each LSTM structure, including the forgetting gate, the input gate, and the output gate. The role of the forgetting gate was to decide what information to discard from the cell state, the input gate decided what new memories were to be stored into the cell state, and the output gate was mainly responsible for the final output of the cell state. The LSTM network protects and controls the cell state through these three gates, realizing the long term remembering, forgetting, and updating of the state. The internal processing of the LSTM was computed as follows:

(4) $$f_{t}=\sigma \left(W_{fh}h_{t-1}+W_{fx}x_{t}+b_{f}\right)$$

(5) $$i_{t}=\sigma \left(W_{ih}h_{t-1}+W_{ix}x_{t}+b_{i}\right)$$

(6) $${\tilde{c}}_{t}=\tanh\left(W_{\tilde{c}h}h_{t-1}+W_{\tilde{c}x}x_{t}+b_{\tilde{c}}\right)$$

(7) $$c_{t}=f_{t}.c_{t-1}+i_{t}.{\overset{\sim }{c}}_{t}$$

(8) $$o_{t}=\sigma \left(W_{oh}h_{t-1}+W_{ox}x_{t}+b_{o}\right)$$

(9) $$h_{t}=o_{t}\cdot \tanh\left(c_{t}\right)$$

In Equations (4)–(9), $f_{t}$*,* $i_{t},$ and $o_{t}$ represent the forgetting gate, input gate and output gate of the LSTM structure, respectively; *c_t_* is the internal state to save the important information extracted from 0~*t* time, which is the carrier of the long-term memory; *h_t_* is the hidden state as the output of the short and long term memory network in the moment *t*, which is the carrier of the short-term memory; [$W_{fx}$, $W_{fh}$], [$W_{ix}$, $W_{ih}$], [$W_{ox}$, $W_{oh}$], and [$W_{\tilde{c}x}{,\;W}_{\tilde{c}h}$] are the weights of the forgetting gate, the input gate, the output gate, and the unitary inputs, respectively; $b_{f}$, $b_{i}$, $b_{o},$ and $b_{\tilde{c}}$ are the biases of the forgetting gate, the input gate, the output gate, and the unitary input, respectively, and *σ* denotes the sigmoid activation function.

Finally, the visually induced motion sickness states (resting, light sickness, heavy sickness) were categorized using fully connected and SoftMax layers, and the proposed model was trained and tested using the 5-fold cross-validation method.

This experiment used Adam optimizer, which can effectively mitigate the overfitting issue during the learning process, and set the learning rate to 1 × 10^−3^. The Adam optimizer combines the stochastic gradient descent algorithm and the adaptive learning rate algorithm, which can quickly converge and reduce the training time, while the Adam optimizer does not need to manually adjust the size of the learning rate and can adaptively adjust the learning rate of each parameter to improve the model and the model’s convergence speed and generalization ability. The experiments also use the Cross Entropy Loss (CEL) function to increase the model’s learning rate to prevent the gradient from disappearing. For each training session, the number of epoch iterations is set to 50, and the batch size is set to 32. In addition, if the loss on the test dataset does not decrease for more than ten consecutive sessions, the training is stopped using the Early Stopping technique, which prevents the overfitting problem caused by the training data.

### 2.7. Performance Evaluation Methods

To assess the effectiveness of the model in this study, a rigorous 5-fold cross-validation approach was employed, i.e., dividing the sample set into five parts and using four of them separately as the training set and the remaining one as the test set. Accuracy was the most commonly used assessment metric to verify the effectiveness of a model, i.e., by measuring the proportion of samples correctly predicted by the model. Additionally, a confusion matrix is a measure of the accuracy of a classification model. Each column of the confusion matrix represents the prediction category, and each row represents the real category of the data. Finally, the recall rate was employed as an additional metric to evaluate the model’s classification performance, offering a comprehensive understanding of its effectiveness.

(10) $$Accuracy=\frac{TP+TN}{TP+FN+FP+TN}$$

(11) $$Recall=\frac{TP}{TP+FN}$$

where *TP* indicates that both the sample and the prediction result are positive, i.e., True Positive (*TP*); *FP* indicates that the sample is negative and the prediction result is positive, i.e., False Positive (*FP*); *TN* indicates that both the sample and the prediction result are negative, i.e., True Negative (*TN*); *FN* means that the sample is positive and the prediction result is negative, i.e., False Negative (*FN*).

## 3. Results and Discussion

### 3.1. SSQ Analysis

**Figure 5:**
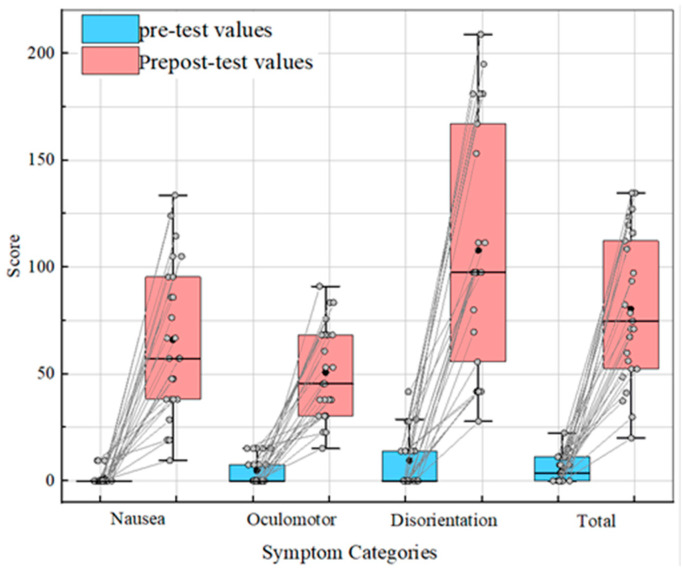
Scores in each category before and after the experiment.

There were 25 subjects in this experiment, including 20 males, aged 21.32 ± 2.27 years, and, according to the statistical results analysis, there was no significant difference between age and gender on the SSQ. According to the pre-test and post-test SSQ scores, the box line diagram of the statistical scores for each weight is shown in Figure 5.

A paired samples t-test revealed significant differences in eye discomfort (t = −7.845, *p* ≤ 0.001) and disorientation (t = −9.715, *p* < 0.001), but not in nausea (N) (t = −1.24, *p* = 0.227). The reason was that in the experimental design, the subjects were required to report immediately and terminate the experiment once they became intolerable, instead of everyone testing for the same duration.

However, the results of the mean SSQ scores before and after the experiment were significantly different (t = −10.17, *p* < 0.001), indicating that the subjects did experience visually induced motion sickness during the task state. This conclusion was consistent with the one found by Sungu Nam et al. [31]. Therefore, the grade of motion sickness can be classified according to the time of the subjects’ oral report.

### 3.2. Functional Connectivity Matrix

Based on the EEG signal preprocessing and the PLV functional connectivity matrix construction methods above, we successfully generated 1500 52 × 52 PLV functional connectivity matrices encompassing the three VISM states. The PLV functional connectivity matrices for the three VISM states of 25 subjects were averaged under six different frequency bands to obtain the respective average PLV functional connectivity matrix plots, as shown in Table 1. Each row and column in the matrix plot corresponds to an electrode channel, and each element represents the average PLV functional connectivity value from the horizontal coordinate electrode channel to the vertical coordinate electrode channel.

As evident from Table 1, notable differences exist in the PLV functional connectivity matrices across different frequency bands. Specifically, the PLV values were overall higher in the delta band and lower in the gamma band. In addition, the PLV functional connectivity matrices for the three states in the same frequency band show similar synchronization patterns but have different synchronization levels.

**Figure 6:**
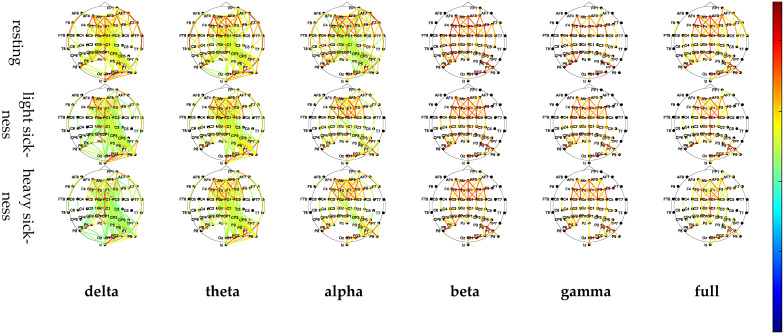
Network topology in six bands and three states.

To facilitate a more intuitive observation of the changes in the brain network topology map of the subjects, the average PLV functional connectivity matrix in the three VIMS states of the six frequency bands was converted into a network topology map, and the results are shown in Figure 6. In the network topology diagram, the lines represent the edges of the network; the color of the lines represents the strength of the connection between the electrodes; the darker the color of the connection lines, the stronger the connection between the two electrodes, and the lighter the color, the weaker the connectivity. In order to facilitate clear observation, the sparsity threshold is set to 0.55.

As Figure 6 illustrates, certain brain regions within the frontal lobe and the center exhibited certain strength of functional connections, but there were obvious differences in connection strength. In addition, the number of functional connections varied across different frequency bands, with the number of functional connections in the delta and theta frequency bands being higher.

From the analysis of network topology maps in the delta and theta bands, it can be observed that the right brain regions were more tightly connected relative to the left brain regions, and the number of brain functional connections was higher in the right central, right parietal, and right occipital lobes relative to the left central, left parietal, and left occipital lobes. As the level of VIMS increases, the temporal lobe experiences an increase in the number of functional connections, albeit with varying degrees of reduced connection strength. In the alpha band, the resting-state cerebral functional connections was greater, and all electrodes in the whole brain showed some strength of functional connections, but some electrode functional connections in the temporal lobe disappeared in the light and heavy halo states. In the beta and gamma frequency bands, the number and strength of functional connections in the left and right brain regions in different VIMS states were relatively balanced. When the subjects’ dizziness degree was higher, the number of functional connections decreased. After VIMS, the brain functional connections were mainly concentrated in the prefrontal lobe. In the full frequency band, as the VIMS level rose, the number of brain functional connections decreased, and the connectivity of the parietal lobe and occipital lobe weakened. In addition, in the resting state, the right temporal lobe and right frontal lobe functional connection was strong, while in the halo state, this functional connection disappeared. Overall, the frontal and temporal lobes were more involved in motion sickness symptoms and were the brain regions that were more sensitive to changes in the VIMS state.

### 3.3. Classified Evaluation

**Figure 7:**
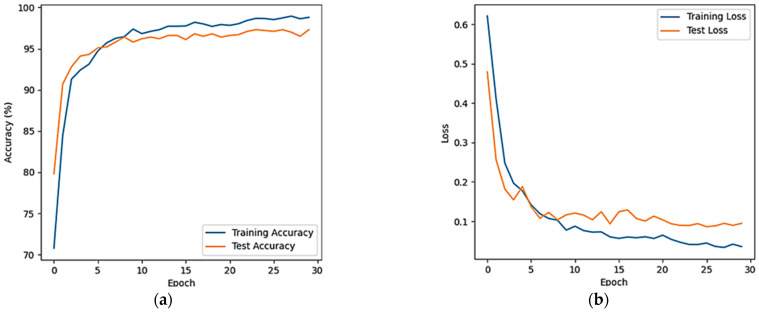
CNN-LSTM model binary classification curve plot. (**a**) Average accuracy; (**b**) average loss ratio.

To observe the classification performance of the proposed model, the average accuracy and average loss rate curves for dichotomous classification (resting, motion sickness) after the five-fold cross-validation of the full-band EEG signals of 25 subjects were plotted, as shown in Figure 7.

As can be seen in Figure 7a, the average accuracy increases dramatically from 1 to 10 epochs on both the training and test sets, and then levels off. The average accuracy on the test set finally stabilizes at around 95%. In Figure 7b, it can be seen that the average loss rate decreases sharply from 1 to 10 epochs, and finally stabilizes at around 0.1% on the test set.

**Figure 8:**
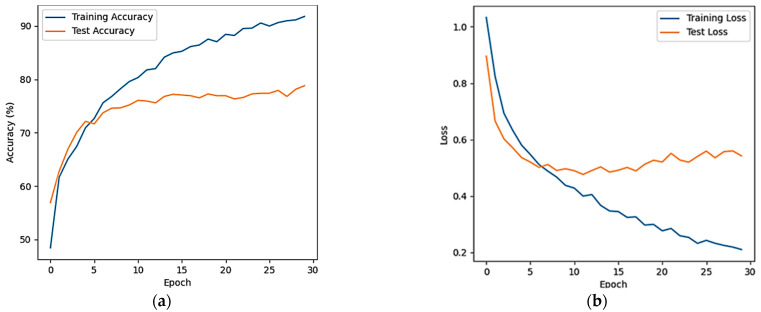
Three classification curves of the CNN-LSTM model. (**a**) Average accuracy; (**b**) average loss ratio.

Figure 8 shows the average accuracy and average loss rate curves for the three classifications (resting, light halo, heavy halo) of the full-band EEG signals from 25 subjects after the CNN-LSTM model. Figure 8a shows the average accuracy of the model and Figure 8b shows the average loss rate.

From Figure 8, it is evident that the average accuracy on the training set steadily rises during the first 30 epochs, paralleled by a consistent decrease in the average loss rate. On the test set, the average accuracy experiences a sharp increase from 1 to 5 epochs, subsequently stabilizing around 80%, while the average loss rate eventually converges to approximately 0.5.

In the VIMS state classification task, by analyzing the changes in the average accuracy and average loss rate of the CNN-LSTM model for both binary and tertiary classification, the model can gradually learn better features during the training and testing process, and achieves a relatively stable and acceptable level of performance on the testing set.

To assess the model’s average accuracy and loss rate compared to other models, the CNN-LSTM model is compared with the SVM model, the CNN model, and the LSTM model, respectively, for the five-fold cross validation classification on this dataset. In this context, the CNN-1, CNN-2, and CNN-3 models represent CNN models of 1-layer, 2-layer and 3-layer convolutional layer, respectively. After the training and testing are carried out several times in the sub-models and frequency bands, the respective average accuracy and standard deviation are obtained, and it can be seen that the average recognition ability and stability of the proposed models are better, and the results are shown in Table 2.

In VIMS classification utilizing the PLV brain functional connectivity dataset, comparing the classification results of all models for the six frequency bands, a comparative analysis of the classification results across all models for the six frequency bands revealed that the gamma band yielded the most optimal outcomes, closely followed by the beta band. It indicates that high-frequency signals (15–45 Hz) perform better on the task of visually induced motion sickness state classification, and show consistency in the classification results across models.

The classification accuracy reported in Table 2 clearly demonstrates the superiority of the CNN-LSTM model over other approaches for both binary and triple classification tasks. Specifically, on the full band, the CNN-LSTM model achieves a maximum average classification accuracy of 97.09% for binary classification and 78.60% for triple classification. Furthermore, when focusing on the gamma band, the maximum average classification accuracy using the CNN-LSTM model is 99.56% for binary classification and 86.94% for triple classification, which is at least 0.16% and 2.34% higher than that of other models, respectively. Meanwhile, a low standard deviation is obtained after five-fold cross-validation on six frequency bands using the model, which verifies the stability and robustness of the model. Thus, it can be demonstrated that the CNN-LSTM model can extract both spatial and temporal features of the data to improve the classification accuracy of visually induced motion sickness with high stability.

In addition, as evident from the three classification results represented in Table 2, among the three CNN models differing in the number of layers, the convolutional layer with two layers has the highest average accuracy and a small standard deviation, which is also the basis for selecting two convolutional layers in the CNN-LSTM model proposed in this paper.

**Figure 9:**
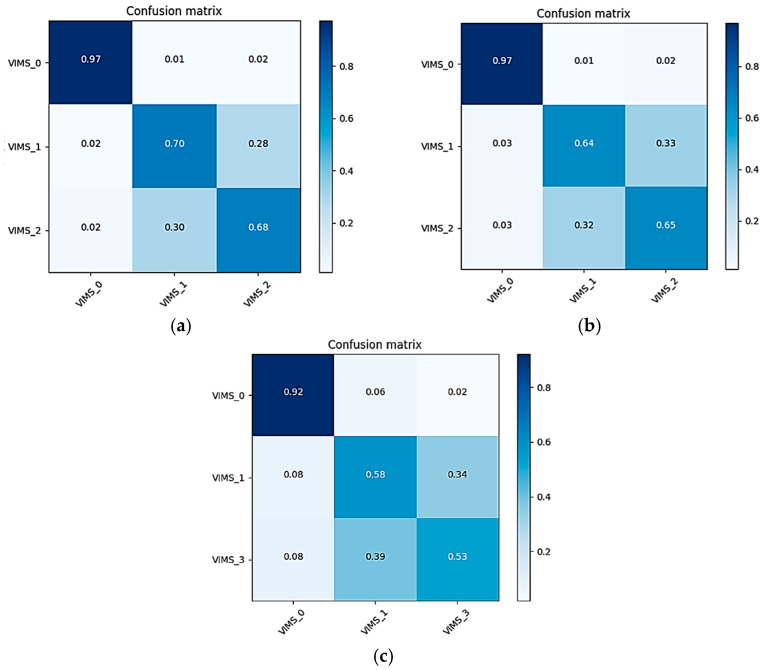
Average confusion matrices for various models. (**a**) CNN-LSTM model average confusion matrix. (**b**) CNN-2 model average confusion matrix. (**c**) Mean confusion matrix for SVM models.

To further evaluate the performance of the three models SVM, CNN-2, and CNN-LSTM for VIMS triple classification, the average confusion matrices of the three models were computed on the full-band EEG signals of the 25 subjects and normalized to the confusion matrix, as shown in Figure 9. The sum of all probabilities in each row is 1, so the diagonal elements represent the recall of each category in turn.

As depicted in Figure 9, the CNN-LSTM model obtained the highest recall across all three classification states, which were 0.97, 0.70, and 0.68, respectively. Comparatively, the CNN-2 model had a recall of 0.97, 0.64, and 0.65, and the SVM model had a recall of 0.92, 0.58, and 0.53 in the three states, respectively. Notably, all three models exhibited the highest recognition rate in the resting state. The CNN-LSTM model and the SVM model have higher recognition rates for both the mild motion sickness category than the severe motion sickness category, and the CNN-2 model has similar recognition rates for both the mild and severe motion sickness categories. Therefore, this result can also further support the aforementioned conclusion of higher classification accuracy for distinguishing the presence or absence of motion sickness. In addition it indicates that the subjects had a higher degree of difficulty in recognizing the mild and severe motion sickness categories.

In order to further verify the classification superiority of the combination method of the brain functional connectivity network and the CNN-LSTM model, this paper compares the classification accuracy of this method with other motion sickness classification methods proposed in other studies, and the comparison results are shown in Table 3. As can be seen from Table 3, the CNN-LSTM model proposed in this paper achieved the highest recognition accuracy in both the binary classification task and the tri-classification task of the visually induced motion sickness state, and has better applicability for VIMS.

This paper proposes a novel CNN-LSTM model for predicting visually induced motion sickness (VIMS), which leverages the spatial feature extraction capabilities of the CNN layers combined with the temporal modeling strengths of the LSTM layers. This model is capable of capturing local features while maintaining long-term dependencies when processing EEG signals. By integrating the model with a brain functional connectivity network, it achieves excellent classification performance. Using the PLV brain functional connectivity network as input features, the CNN-LSTM model was compared with the SVM, standalone CNN, and LSTM models for the binary and ternary classification of VIMS states in terms of average accuracy and standard deviation. The CNN-LSTM network achieved a maximum average binary classification accuracy of 99.56% and a maximum ternary classification accuracy of 86.94%. Compared to other models, the proposed model showed at least a 0.16% increase in average accuracy for binary classification tasks and at least a 2.14% increase for ternary classification tasks, along with lower standard deviations, indicating better robustness and reliability. Furthermore, by comparing the average confusion matrices of the SVM model, the CNN model, and the CNN-LSTM model, the proposed model achieved the highest recall rates for the states of rest, mild discomfort, and severe discomfort, at 0.97, 0.70, and 0.68, respectively, further demonstrating the superiority of this deep learning classification model.

## 4. Conclusions

In this paper, a comprehensive analysis of EEG data was conducted to explore the EEG functional connectivity across various frequency bands under different VIMS states. Subsequently, a classification model for VIMS was developed, leveraging the PLV functional connectivity matrix in conjunction with the architecture of CNN-LSTM. This approach enabled the accurate classification of VISM states, yielding promising results. However, due to the relatively small sample size, the performance of the model should be further tested in a large sample. In addition, this paper only briefly analyzes the scalp EEG signals, and the network structure of the cerebral cortex can be further investigated in the future through the source localization technique [36], which can provide an effective way for the auxiliary diagnosis of motion sickness disorder (MSD), help with early prevention for patients with MSD, and promote the wider application of virtual reality technology.

## Figures and Tables

**Table 1:** Average PLV functional connectivity matrix chart for six frequency bands.

	Delta	Theta	Alpha	Beta	Gamma	Full	
resting	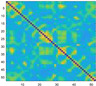	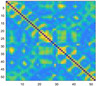	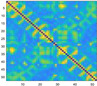	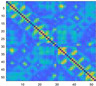	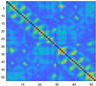	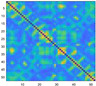	
light sickness	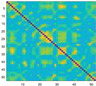	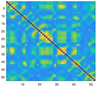	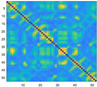	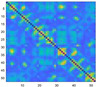	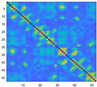	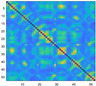	
heavy sickness	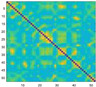	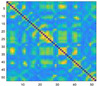	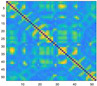	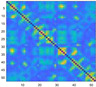	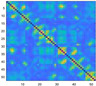	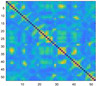	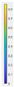

**Table 2:** Comparison of classification accuracy of different models.

Model	Two-Class Average Accuracy ± Standard Deviation (%)				Three-Class Average Accuracy ± Standard Deviation (%)					
	SVM	CNN-2	LSTM	CNN-LSTM	SVM	CNN-1	CNN-2	CNN-3	LSTM	CNN-LSTM
Full	94.48 ± 1.40	95.00 ± 1.30	85.71 ± 2.88	97.09 ± 0.80	72.80 ± 2.14	74.60 ± 3.59	76.46 ± 3.31	61.73 ± 2.58	70.78 ± 1.58	78.60 ± 1.48
Delta	77.80 ± 1.81	82.48 ± 1.96	75.03 ± 2.24	82.79 ± 1.03	56.13 ± 2.58	60.27 ± 2.21	61.80 ± 2.07	52.34 ± 3.24	50.67 ± 3.12	64.27 ± 0.92
Theta	89.52 ± 1.76	92.44 ± 2.02	88.83 ± 3.42	93.20 ± 1.81	61.33 ± 2.99	61.20 ± 2.39	62.67 ± 2.64	57.47 ± 3.45	55.72 ± 2.27	68.14 ± 2.22
Alpha	90.63 ± 3.15	91.48 ± 2.88	89.32 ± 1.58	95.10 ± 1.16	64.67 ± 2.76	66.33 ± 3.50	67.59 ± 3.30	60.46 ± 2.46	58.96 ± 2.75	71.74 ± 2.60
Beta	98.78 ± 0.64	97.99 ± 0.83	92.27 ± 1.46	98.96 ± 0.45	76.07 ± 2.58	80.20 ± 1.94	80.86 ± 1.71	64.13 ± 2.31	63.27 ± 3.93	83.94 ± 2.43
Gamma	98.94 ± 0.59	99.40 ± 0.37	97.56 ± 0.92	99.56 ± 0.34	80.80 ± 2.16	84.32 ± 2.97	84.60 ± 2.34	67.13 ± 3.91	72.70 ± 1.41	86.94 ± 1.38

**Table 3:** Classification accuracy of VIMS recognition based on EEG.

Studies	Feature Extraction	Classification Model	Two-Class Average Accuracy ± Standard Deviation (%)	Three-Class Average Accuracy ± Standard Deviation (%)
Hua et al. [26]	Variational mode decomposition, sample entropy, permutation entropy, center frequency	SVM classifier	98.3%	-
Li et al. [32]	Power spectral density and PCA	Voting classifier	76.3%	-
Liu et al. [33]	Raw EEG data	VIMSNet	96.7%	-
Lin et al. [34]	Power spectrum analysis and PCA	Fuzzy neural network	82%	-
Mawalid et al. [35]	Time domain feature	Naive Bayes classifier	83.8%	-
Zhu et al. [25]	Rhythm wavelet packetEnergy proportion	polynomial-SVM KNN RBF-SVM	79.25% 77.5% 73.83%	68.15% 70.37% 64.07%
This study	Brain functions connect networks	CNN-LSTM	99.56%	86.54%

## Data Availability

The data that support the findings of this work are available upon request.
